# Perioperative do-not-resuscitate orders: it is time to talk

**DOI:** 10.1186/1471-2253-13-1

**Published:** 2013-01-14

**Authors:** Peter G Brindley

**Affiliations:** 1 Division of Critical Care Medicine, University of Alberta, Edmonton, Alberta, Canada

**Keywords:** Do not resuscitate, Perioperative, Cardiopulmonary resuscitation

## Abstract

A study by Burkle *et al.* in *BMC Anesthesiology* examined attitudes around perioperative do-not-resuscitate orders. Questionnaires were given to patients, as well as to anesthesiologists, internists and surgeons. The study has limitations and is open to interpretation. However, the findings are important. There appear to be attitudinal differences between patients and doctors, and between specialties. A small majority of patients are content to have a do-not-resuscitate order postponed during the perioperative period. A large majority expects open communication from doctors before proceeding. However, this article could also encourage a broader debate. This is about how to respect patient autonomy, while ensuring that resuscitation truly serves the patient’s best interests. This commentary outlines how more communication is needed at the bedside and in wider society.

## Background

Cardiopulmonary resuscitation (CPR) can prevent premature death. It can also prolong inevitable death, extend patient suffering, consume scarce resources, and exacerbate staff burnout [1-4]. As a result, CPR may represent the best and worst of acute care medicine. It is also currently the only medical intervention expected for everyone without explicit contrary documentation. It is, therefore, an important topic for both practitioners and patients. A study by Burkle *et al.*[5] in BMC Anesthesiology adds to this discussion, but from an understudied area: the Operating Room (OR).

### Attitudes about perioperative do not resuscitate (DNR) orders

Burkle et al. [5] surveyed 500 patients and 384 doctors regarding their attitudes to perioperative DNR orders. Limitations include the single centre and the reliance upon questionnaires. Therefore, results may not be fully exportable to other jurisdictions, or to busy clinical practice. However, the findings are useful and provocative. Firstly, while over three-quarters of patients knew their resuscitation wishes, only approximately one-half had them recorded (and one-quarter of those under 50-years). Presumably, the first lesson is to increase patient documentation.

Burkle et al. found that 57% of patients believed that a peri-operative DNR should be suspended [5]. This contrasted with only 18% of anesthesiologists, which in turn contrasts with 60% of anesthesiologists from a prior study [6]. The data suggests that attitudinal-gaps exist between care-givers and care-receivers. Moreover, attitudes may have changed over time. Assumptions may also differ between medical specialties. This means that the issue of perioperative DNR has the potential for confusion and conflict. However, precisely because it is a complex topic, it could also broaden our understanding of resuscitation within modern medical practice.

It can be difficult to separate usual anesthesia from some form of resuscitation. Anesthesiologists routinely deliver vasoactive agents, bolus fluids and intubate [7]. This makes it harder to distinguish between ordinary and extra-ordinary anesthetic care. It can also be difficult to separate the intraoperative cardiac arrest that results from end-stage disease, as opposed to surgical or anesthetic iatrogenesis [8]. Therefore, it has been argued that an “OR DNR” is different than a DNR elsewhere [7]. Similarly, an OR death is likely a different kind of death [6]. This may help explain why Burkle found that many clinicians were uncertain how to proceed [5]. This is an issue that needs to be addressed, especially given that approximately 15% of surgical patients have some form of pre-existing DNR [7].

### How to proceed in the setting of a perioperative DNR patient

In contrast, patients seem comparatively clear about how to proceed. For example, Burkle found that an overwhelming majority (92%) expected to be spoken to prior to surgery [5]. It is worth stressing what this means. Meaningful communication is more than just scripted words before anesthetic induction. Optimal communication requires a candid bilateral exchange, an examination of assumptions, and a confirmation of understanding [8]. This means that preoperative communication requires time, patience, and experience. It also requires time to listen. This puts additional pressure on a busy OR.

Communication breakdowns are not uncommon between patients and clinicians. Regardless, if “communication” means “sharing, uniting or making understanding common” [8] then both sides need to invest time and effort. Fortunately, resources and strategies exist for the clinician [9]. Unfortunately, these non-technical skills are not always taught, and are not usually innate [8,9]. It is no longer enough for anesthesiologists to be only technically-proficient [9]. They should also be specialists in peri-operative communication [8]. Expressed another way, “verbal dexterity” should match procedural-dexterity and factual-know-how [8]. Similarly, we should modernize our understanding of resuscitation.

## Discussion

### A more complete understanding of resuscitation

Some practitioners, including Burkle et al., discuss resuscitation as an all-or-none proposition. In contrast, we should separately address interventions such as chest compressions, defibrillation and vasoactive agents [10]. This takes more time but better reflects reality. After all, resuscitation of the pulseless patient is still very unlikely to be successful despite decades of advances. In contrast, resuscitating with a pulse is typically successful. In a typical North American Intensive Care Unit (ICU), approximately 80% of all-comers will survive to discharge. This compares with only approximately 30% if CPR is required, and only 10% if that CPR includes chest compressions [3].

Over six decades, CPR has metamorphosed from “occasional” to “typical” to “expected” [2]. This is also worth discussing. All medical innovations are presumably conceived with noble intentions and intended only for select patients. However, the history of CPR shows how indications expand, even when budgets do not. None of CPR’s originators argued for it to be universal [11-13]. However, we now perform chest compressions on essentially anyone that insists [2-4]. This means that autonomy is respected. It also means that compressions are no longer justified in a manner expected of treatments that are invasive, expensive, and typically unsuccessful. Despite consistent predictors of poor survival, we have nearly one million annual attempts in North America, and one billion dollars that might benefit patients elsewhere [1,2].

### The reality of cardiopulmonary resuscitation

In the developed world, approximately 70% of deaths now occur in-hospital, and 25% of these in ICU [14,15]. Therefore, death is increasingly an institutionalized and technologically-supported phenomenon [15]. Universal CPR leads to universal ICU admission because post-arrest patients cannot be managed elsewhere. This helps explain why approximately 1% of U.S. GDP and 20-30% of the hospital budget is now spent on ICU [16,17]. It also means that resuscitation discussions often occur under pressure and between families and physicians who are unfamiliar with each other [7].

Burkle lauded that only 1/3^rd^ as many anesthesiologists would unilaterally suspend a DNR compared to a 1994 study [5,6]. Perhaps autonomy now supersedes all other considerations. However, there are other possibilities. Possibly, our definition of progress means that we do more but never less. Perhaps it is the fear of litigation, or perhaps that is an excuse to avoid lengthy or contentious discussions. Maybe it is simply easier to think in binary terms i.e. do everything or do nothing. Regardless, this author worries that some doctors no longer feel authorized to stand by unpopular but considered opinions.

## Conclusion

Obviously resuscitation should be individualized, and obviously our ability to predict is imperfect. However, if we fail to communicate properly then we become mere technicians who perform- but do not refuse- interventions, and who start- but do not stop- machines. We have failed to communicate that it is not technically difficult to maintain some patients beyond any likelihood of leaving hospital. In addition, the majority of patients do not die because we cannot keep their heart and lungs going [14,15]. When we do stop it is not because of no other option, but rather because it is time. Physicians must advocate, but not solely for more resources. We also need to advocate for time to talk, and to listen.

## Competing interest

The author declares that he has no competing interests.

## Authors’ contribution

PB read and approved the final manuscript. PB drafted, revised and approved the manuscript. All authors read and approved the final manuscript.

## Pre-publication history

The pre-publication history for this paper can be accessed here:

http://www.biomedcentral.com/1471-2253/13/1/prepub
